# 3,4,5-Trihydr­oxy-*N*′-[(1-methyl-1*H*-indol-2-yl)methyl­idene]benzohydrazide

**DOI:** 10.1107/S1600536809027032

**Published:** 2009-07-18

**Authors:** Hamid Khaledi, Siti Munirah Saharin, Hapipah Mohd Ali, Ward T. Robinson, Mahmood A. Abdulla

**Affiliations:** a Department of Chemistry, University of Malaya, 50603 Kuala Lumpur, Malaysia; b Department of Molecular Medicine, University of Malaya, 50603 Kuala Lumpur, Malaysia

## Abstract

The structure of the title compound, C_17_H_15_N_3_O_4_, displays inter­molecular O—H⋯N and O—H⋯O hydrogen bonding between adjacent mol­ecules. Intra­molecular O—H⋯O hydrogen bonds also occur. The molecule is essentially planar with a deviation of 0.090 (1) Å from the best plane running through the connected ring systems.

## Related literature

For related compounds see: Khaledi *et al.* (2008*a* ▶,*b* ▶, 2009 ▶).
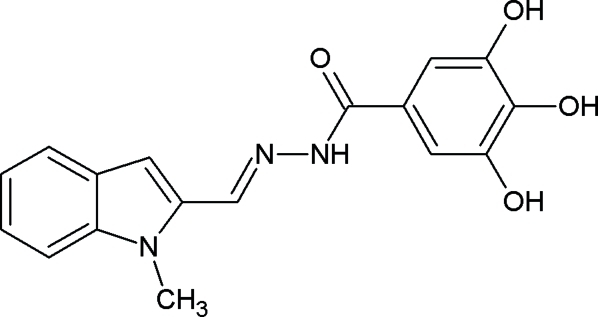


## Experimental

### 

#### Crystal data

C_17_H_15_N_3_O_4_
*M* *_r_* = 325.32
Monoclinic, 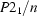

*a* = 9.0839 (2) Å
*b* = 13.1684 (3) Å
*c* = 12.4414 (3) Å
β = 104.2740 (10)°
*V* = 1442.30 (6) Å^3^
*Z* = 4
Mo *K*α radiation
μ = 0.11 mm^−1^
*T* = 100 K
0.49 × 0.16 × 0.09 mm

#### Data collection

Bruker APEXII CCD area-detector diffractometer
Absorption correction: multi-scan (*SADABS*; Sheldrick, 1996 ▶) *T*_min_ = 0.948, *T*_max_ = 0.991
10177 measured reflections
4070 independent reflections
3153 reflections with *I* > 2σ(*I*)
*R*_int_ = 0.019

#### Refinement

*R*[*F* ^2^ > 2σ(*F* ^2^)] = 0.044
*wR*(*F* ^2^) = 0.127
*S* = 0.99
4070 reflections
221 parameters
H-atom parameters constrained
Δρ_max_ = 0.63 e Å^−3^
Δρ_min_ = −0.26 e Å^−3^

### 

Data collection: *APEX2* (Bruker, 2007 ▶); cell refinement: *APEX2*; data reduction: *SAINT* (Bruker, 2007 ▶); program(s) used to solve structure: *SHELXS97* (Sheldrick, 2008 ▶); program(s) used to refine structure: *SHELXL97* (Sheldrick, 2008 ▶); molecular graphics: *X-SEED* (Barbour, 2001 ▶); software used to prepare material for publication: *publCIF* (Westrip, 2009 ▶).

## Supplementary Material

Crystal structure: contains datablocks I, global. DOI: 10.1107/S1600536809027032/hg2532sup1.cif

Structure factors: contains datablocks I. DOI: 10.1107/S1600536809027032/hg2532Isup2.hkl

Additional supplementary materials:  crystallographic information; 3D view; checkCIF report

## Figures and Tables

**Table 1:** Hydrogen-bond geometry (Å, °)

*D*—H⋯*A*	*D*—H	H⋯*A*	*D*⋯*A*	*D*—H⋯*A*
O2—H2*O*⋯O4^i^	0.84	1.80	2.6119 (14)	164
O1—H1*O*⋯N2^i^	0.84	2.06	2.7759 (15)	142
O3—H3*O*⋯O2^ii^	0.84	2.12	2.8469 (14)	144
O1—H1*O*⋯O2	0.84	2.51	2.8570 (14)	106
O3—H3*O*⋯O2	0.84	2.31	2.7560 (14)	113

Symmetry codes: (i) 
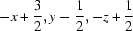
; (ii) 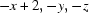.

**Fig. 1.**
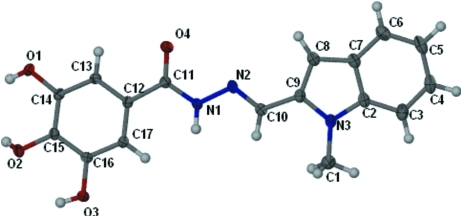
Thermal ellipsoid plot (Barbour, 2001) of the title compound at 70% probability level. Hydrogen atoms are drawn as spheres of arbitrary radius.

## supplementary crystallographic information

### Experimental

A mixture of 1-Methylindole-2-carboxaldehyde (0.80 g, 5 mmol) and 3,4,5-trihydroxybenzoylhydrazine(0.92 g, 5 mmol) in the presence of acetic acid (1 ml) was heated in ethanol (70 ml) for 6 h. The solution was then cooled and filtered to remove the unreacted hydrazine. The filtrate was poured to water (400 ml), the solid product formed were filtered off, washed with diethyl ether,and dried in an oven. Suitable crystals for X-ray analysis were obtained by slow evaporation of an ethanol solution at room temperature.

### Refinement

All Hydrogen atoms were placed at calculated positions (C—H 0.95–0.98, N—H 0.88 and O—H 0.84 Å), with U(H) set to 1.2–1.5 times *U*_eq_(*C,N,O*).

### Crystal data

**Table:**

C_17_H_15_N_3_O_4_	*F*(000) = 680
*M**_r_* = 325.32	*D*_x_ = 1.498 Mg m^−^^3^
Monoclinic, *P*2_1_/*n*	Mo *K*α radiation, λ = 0.71073 Å
Hall symbol: -P 2yn	Cell parameters from 3739 reflections
*a* = 9.0839 (2) Å	θ = 2.3–30.4°
*b* = 13.1684 (3) Å	µ = 0.11 mm^−^^1^
*c* = 12.4414 (3) Å	*T* = 100 K
β = 104.274 (1)°	Block, green
*V* = 1442.30 (6) Å^3^	0.49 × 0.16 × 0.09 mm
*Z* = 4	

### Data collection

**Table:**

Bruker APEXII CCD area-detector diffractometer	4070 independent reflections
Radiation source: fine-focus sealed tube	3153 reflections with *I* > 2σ(*I*)
graphite	*R*_int_ = 0.019
ω scans	θ_max_ = 30.5°, θ_min_ = 2.3°
Absorption correction: multi-scan (*SADABS*; Sheldrick, 1996)	*h* = −9→12
*T*_min_ = 0.948, *T*_max_ = 0.991	*k* = −18→18
10177 measured reflections	*l* = −17→14

### Refinement

**Table:**

Refinement on *F*^2^	Primary atom site location: structure-invariant direct methods
Least-squares matrix: full	Secondary atom site location: difference Fourier map
*R*[*F*^2^ > 2σ(*F*^2^)] = 0.044	Hydrogen site location: inferred from neighbouring sites
*wR*(*F*^2^) = 0.127	H-atom parameters constrained
*S* = 0.99	*w* = 1/[σ^2^(*F*_o_^2^) + (0.0672*P*)^2^ + 0.9156*P*] where *P* = (*F*_o_^2^ + 2*F*_c_^2^)/3
4070 reflections	(Δ/σ)_max_ < 0.001
221 parameters	Δρ_max_ = 0.63 e Å^−^^3^
0 restraints	Δρ_min_ = −0.26 e Å^−^^3^

### Special details

**Table:**

Geometry. All e.s.d.'s (except the e.s.d. in the dihedral angle between two l.s. planes) are estimated using the full covariance matrix. The cell e.s.d.'s are taken into account individually in the estimation of e.s.d.'s in distances, angles and torsion angles; correlations between e.s.d.'s in cell parameters are only used when they are defined by crystal symmetry. An approximate (isotropic) treatment of cell e.s.d.'s is used for estimating e.s.d.'s involving l.s. planes.
Refinement. Refinement of *F*^2^ against ALL reflections. The weighted *R*-factor *wR* and goodness of fit *S* are based on *F*^2^, conventional *R*-factors *R* are based on *F*, with *F* set to zero for negative *F*^2^. The threshold expression of *F*^2^ > σ(*F*^2^) is used only for calculating *R*-factors(gt) *etc*. and is not relevant to the choice of reflections for refinement. *R*-factors based on *F*^2^ are statistically about twice as large as those based on *F*, and *R*- factors based on ALL data will be even larger.

### Fractional atomic coordinates and isotropic or equivalent isotropic displacement parameters (Å^2^)

**Table:**

	*x*	*y*	*z*	*U*_iso_*/*U*_eq_	
N1	0.47487 (13)	0.36671 (9)	0.05923 (10)	0.0132 (2)	
H1N	0.4655	0.3333	−0.0034	0.016*	
N2	0.38051 (13)	0.44784 (9)	0.06479 (10)	0.0134 (2)	
N3	0.06173 (13)	0.56577 (9)	−0.13005 (10)	0.0151 (2)	
O1	0.99391 (12)	0.12533 (8)	0.33141 (8)	0.0165 (2)	
H1O	1.0219	0.0645	0.3313	0.025*	
O2	0.98618 (11)	0.01716 (7)	0.13104 (8)	0.0149 (2)	
H2O	0.9766	−0.0305	0.1737	0.022*	
O3	0.77044 (12)	0.07206 (8)	−0.05744 (8)	0.0189 (2)	
H3O	0.8473	0.0349	−0.0506	0.028*	
O4	0.59664 (13)	0.38379 (8)	0.23996 (8)	0.0199 (2)	
C1	0.04811 (18)	0.51469 (13)	−0.23489 (13)	0.0226 (3)	
H1A	−0.0426	0.5393	−0.2887	0.034*	
H1B	0.0393	0.4413	−0.2247	0.034*	
H1C	0.1383	0.5288	−0.2623	0.034*	
C2	−0.02892 (15)	0.64421 (11)	−0.11137 (12)	0.0155 (3)	
C3	−0.15129 (16)	0.69271 (11)	−0.18421 (13)	0.0192 (3)	
H3	−0.1834	0.6736	−0.2600	0.023*	
C4	−0.22304 (17)	0.76936 (12)	−0.14091 (14)	0.0213 (3)	
H4	−0.3059	0.8039	−0.1883	0.026*	
C5	−0.17667 (17)	0.79760 (11)	−0.02853 (14)	0.0219 (3)	
H5	−0.2291	0.8505	−0.0016	0.026*	
C6	−0.05636 (17)	0.74988 (11)	0.04323 (13)	0.0195 (3)	
H6	−0.0259	0.7692	0.1191	0.023*	
C7	0.02010 (16)	0.67213 (11)	0.00177 (12)	0.0163 (3)	
C8	0.14341 (16)	0.60759 (11)	0.05098 (12)	0.0162 (3)	
H8	0.1997	0.6085	0.1263	0.019*	
C9	0.16628 (15)	0.54349 (10)	−0.03077 (11)	0.0137 (3)	
C10	0.27384 (15)	0.46201 (10)	−0.02389 (11)	0.0134 (3)	
H10	0.2662	0.4180	−0.0856	0.016*	
C11	0.58127 (15)	0.33924 (10)	0.15038 (11)	0.0129 (3)	
C12	0.68091 (14)	0.25165 (10)	0.13972 (11)	0.0123 (3)	
C13	0.78798 (15)	0.22437 (10)	0.23633 (11)	0.0132 (3)	
H13	0.7917	0.2597	0.3035	0.016*	
C14	0.88907 (15)	0.14590 (10)	0.23472 (11)	0.0128 (3)	
C15	0.88441 (15)	0.09305 (10)	0.13689 (11)	0.0122 (2)	
C16	0.77684 (15)	0.12083 (10)	0.04008 (11)	0.0135 (3)	
C17	0.67475 (15)	0.19925 (10)	0.04105 (11)	0.0143 (3)	
H17	0.6014	0.2171	−0.0249	0.017*	

### Atomic displacement parameters (Å^2^)

**Table:**

	*U*^11^	*U*^22^	*U*^33^	*U*^12^	*U*^13^	*U*^23^
N1	0.0140 (5)	0.0112 (5)	0.0140 (5)	0.0024 (4)	0.0029 (4)	−0.0013 (4)
N2	0.0120 (5)	0.0110 (5)	0.0173 (5)	0.0015 (4)	0.0040 (4)	0.0006 (4)
N3	0.0132 (5)	0.0141 (5)	0.0174 (6)	0.0019 (4)	0.0028 (4)	0.0011 (4)
O1	0.0180 (5)	0.0172 (5)	0.0121 (4)	0.0047 (4)	−0.0006 (4)	0.0011 (4)
O2	0.0183 (5)	0.0122 (5)	0.0157 (5)	0.0049 (4)	0.0069 (4)	0.0039 (4)
O3	0.0196 (5)	0.0214 (5)	0.0139 (5)	0.0075 (4)	0.0010 (4)	−0.0046 (4)
O4	0.0232 (5)	0.0203 (5)	0.0151 (5)	0.0068 (4)	0.0027 (4)	−0.0035 (4)
C1	0.0219 (7)	0.0248 (8)	0.0192 (7)	0.0035 (6)	0.0014 (6)	−0.0027 (6)
C2	0.0135 (6)	0.0124 (6)	0.0219 (7)	0.0001 (5)	0.0066 (5)	0.0025 (5)
C3	0.0148 (6)	0.0182 (7)	0.0247 (7)	0.0009 (5)	0.0050 (5)	0.0049 (6)
C4	0.0151 (6)	0.0168 (7)	0.0325 (8)	0.0031 (5)	0.0070 (6)	0.0071 (6)
C5	0.0185 (7)	0.0134 (6)	0.0370 (9)	0.0020 (5)	0.0128 (6)	0.0013 (6)
C6	0.0193 (7)	0.0154 (7)	0.0263 (8)	−0.0008 (5)	0.0101 (6)	−0.0018 (5)
C7	0.0149 (6)	0.0129 (6)	0.0220 (7)	−0.0004 (5)	0.0068 (5)	0.0009 (5)
C8	0.0155 (6)	0.0142 (6)	0.0197 (7)	0.0003 (5)	0.0061 (5)	0.0004 (5)
C9	0.0122 (6)	0.0123 (6)	0.0165 (6)	−0.0002 (5)	0.0034 (5)	0.0022 (5)
C10	0.0125 (6)	0.0126 (6)	0.0155 (6)	−0.0008 (5)	0.0042 (5)	−0.0007 (5)
C11	0.0134 (6)	0.0121 (6)	0.0135 (6)	0.0001 (5)	0.0042 (5)	0.0011 (5)
C12	0.0122 (6)	0.0116 (6)	0.0131 (6)	0.0007 (4)	0.0031 (5)	0.0013 (5)
C13	0.0148 (6)	0.0131 (6)	0.0120 (6)	0.0008 (5)	0.0034 (5)	−0.0004 (5)
C14	0.0131 (6)	0.0132 (6)	0.0117 (6)	0.0001 (5)	0.0022 (4)	0.0029 (5)
C15	0.0120 (6)	0.0111 (6)	0.0142 (6)	0.0012 (4)	0.0042 (5)	0.0020 (5)
C16	0.0147 (6)	0.0142 (6)	0.0120 (6)	0.0006 (5)	0.0038 (5)	−0.0016 (5)
C17	0.0142 (6)	0.0147 (6)	0.0129 (6)	0.0025 (5)	0.0013 (5)	0.0007 (5)

### Geometric parameters (Å, °)

**Table:**

N1—C11	1.3455 (17)	C4—C5	1.407 (2)
N1—N2	1.3821 (15)	C4—H4	0.9500
N1—H1N	0.8800	C5—C6	1.380 (2)
N2—C10	1.2900 (17)	C5—H5	0.9500
N3—C2	1.3760 (18)	C6—C7	1.405 (2)
N3—C9	1.3912 (17)	C6—H6	0.9500
N3—C1	1.4455 (19)	C7—C8	1.419 (2)
O1—C14	1.3647 (16)	C8—C9	1.376 (2)
O1—H1O	0.8400	C8—H8	0.9500
O2—C15	1.3755 (16)	C9—C10	1.4394 (19)
O2—H2O	0.8400	C10—H10	0.9500
O3—C16	1.3613 (16)	C11—C12	1.4921 (18)
O3—H3O	0.8400	C12—C13	1.3941 (18)
O4—C11	1.2365 (17)	C12—C17	1.3972 (18)
C1—H1A	0.9800	C13—C14	1.3859 (18)
C1—H1B	0.9800	C13—H13	0.9500
C1—H1C	0.9800	C14—C15	1.3936 (18)
C2—C3	1.403 (2)	C15—C16	1.3995 (18)
C2—C7	1.416 (2)	C16—C17	1.3900 (19)
C3—C4	1.381 (2)	C17—H17	0.9500
C3—H3	0.9500		
			
C11—N1—N2	119.41 (11)	C6—C7—C8	133.41 (14)
C11—N1—H1N	120.3	C2—C7—C8	107.14 (12)
N2—N1—H1N	120.3	C9—C8—C7	107.18 (13)
C10—N2—N1	114.41 (11)	C9—C8—H8	126.4
C2—N3—C9	108.32 (12)	C7—C8—H8	126.4
C2—N3—C1	125.46 (12)	C8—C9—N3	109.41 (12)
C9—N3—C1	126.22 (12)	C8—C9—C10	129.58 (13)
C14—O1—H1O	109.5	N3—C9—C10	120.98 (12)
C15—O2—H2O	109.5	N2—C10—C9	120.95 (13)
C16—O3—H3O	109.5	N2—C10—H10	119.5
N3—C1—H1A	109.5	C9—C10—H10	119.5
N3—C1—H1B	109.5	O4—C11—N1	122.01 (12)
H1A—C1—H1B	109.5	O4—C11—C12	120.71 (12)
N3—C1—H1C	109.5	N1—C11—C12	117.28 (12)
H1A—C1—H1C	109.5	C13—C12—C17	119.91 (12)
H1B—C1—H1C	109.5	C13—C12—C11	115.60 (12)
N3—C2—C3	130.13 (14)	C17—C12—C11	124.48 (12)
N3—C2—C7	107.95 (12)	C14—C13—C12	120.23 (12)
C3—C2—C7	121.92 (13)	C14—C13—H13	119.9
C4—C3—C2	117.12 (14)	C12—C13—H13	119.9
C4—C3—H3	121.4	O1—C14—C13	117.13 (12)
C2—C3—H3	121.4	O1—C14—C15	122.35 (12)
C3—C4—C5	121.76 (14)	C13—C14—C15	120.49 (12)
C3—C4—H4	119.1	O2—C15—C14	122.20 (12)
C5—C4—H4	119.1	O2—C15—C16	118.70 (12)
C6—C5—C4	121.17 (14)	C14—C15—C16	119.04 (12)
C6—C5—H5	119.4	O3—C16—C17	118.44 (12)
C4—C5—H5	119.4	O3—C16—C15	120.73 (12)
C5—C6—C7	118.60 (15)	C17—C16—C15	120.83 (12)
C5—C6—H6	120.7	C16—C17—C12	119.49 (12)
C7—C6—H6	120.7	C16—C17—H17	120.3
C6—C7—C2	119.43 (13)	C12—C17—H17	120.3
			
C11—N1—N2—C10	−173.94 (12)	C8—C9—C10—N2	−9.1 (2)
C9—N3—C2—C3	−179.04 (14)	N3—C9—C10—N2	172.91 (13)
C1—N3—C2—C3	0.1 (2)	N2—N1—C11—O4	0.8 (2)
C9—N3—C2—C7	0.29 (15)	N2—N1—C11—C12	−179.57 (11)
C1—N3—C2—C7	179.39 (13)	O4—C11—C12—C13	0.73 (19)
N3—C2—C3—C4	179.14 (14)	N1—C11—C12—C13	−178.95 (12)
C7—C2—C3—C4	−0.1 (2)	O4—C11—C12—C17	−177.78 (13)
C2—C3—C4—C5	−0.4 (2)	N1—C11—C12—C17	2.5 (2)
C3—C4—C5—C6	0.3 (2)	C17—C12—C13—C14	0.5 (2)
C4—C5—C6—C7	0.2 (2)	C11—C12—C13—C14	−178.13 (12)
C5—C6—C7—C2	−0.7 (2)	C12—C13—C14—O1	177.99 (12)
C5—C6—C7—C8	−178.83 (15)	C12—C13—C14—C15	−0.4 (2)
N3—C2—C7—C6	−178.74 (13)	O1—C14—C15—O2	−0.6 (2)
C3—C2—C7—C6	0.7 (2)	C13—C14—C15—O2	177.66 (12)
N3—C2—C7—C8	−0.15 (16)	O1—C14—C15—C16	−177.82 (12)
C3—C2—C7—C8	179.24 (13)	C13—C14—C15—C16	0.4 (2)
C6—C7—C8—C9	178.27 (16)	O2—C15—C16—O3	1.6 (2)
C2—C7—C8—C9	−0.04 (16)	C14—C15—C16—O3	178.93 (12)
C7—C8—C9—N3	0.22 (16)	O2—C15—C16—C17	−177.94 (12)
C7—C8—C9—C10	−177.98 (14)	C14—C15—C16—C17	−0.6 (2)
C2—N3—C9—C8	−0.32 (16)	O3—C16—C17—C12	−178.85 (12)
C1—N3—C9—C8	−179.42 (13)	C15—C16—C17—C12	0.7 (2)
C2—N3—C9—C10	178.06 (12)	C13—C12—C17—C16	−0.6 (2)
C1—N3—C9—C10	−1.0 (2)	C11—C12—C17—C16	177.82 (13)
N1—N2—C10—C9	179.93 (12)		

### Hydrogen-bond geometry (Å, °)

**Table:**

*D*—H···*A*	*D*—H	H···*A*	*D*···*A*	*D*—H···*A*
O2—H2O···O4^i^	0.84	1.80	2.6119 (14)	164
O1—H1O···N2^i^	0.84	2.06	2.7759 (15)	142
O3—H3O···O2^ii^	0.84	2.12	2.8469 (14)	144
O1—H1O···O2	0.84	2.51	2.8570 (14)	106
O3—H3O···O2	0.84	2.31	2.7560 (14)	113

Symmetry codes: (i) −*x*+3/2, *y*−1/2, −*z*+1/2; (ii) −*x*+2, −*y*, −*z*.
